# New Insight Regarding the Relationship Between Enantioselective Toxicity Difference and Enantiomeric Toxicity Interaction from Chiral Ionic Liquids

**DOI:** 10.3390/ijms20246163

**Published:** 2019-12-06

**Authors:** Huilin Ge, Min Zhou, Daizhu Lv, Mingyue Wang, Cunzhu Dong, Yao Wan, Zhenshan Zhang, Suru Wang

**Affiliations:** 1 Hainan Key Laboratory of Tropical Fruit and Vegetable Products Quality and Safety, Analysis and Testing Center, Chinese Academy of Tropical Agricultural Sciences, Haikou 571101, China; zhoumin05@yeah.net (M.Z.); ldz162000@126.com (D.L.); wanyao_analyt@163.com (Y.W.); zhenshanzhang@163.com (Z.Z.); wsr28412466@163.com (S.W.); 2 College of Plant Protection, Hainan University, Haikou 570228, China; czd@hainu.edu.cn

**Keywords:** chiral ionic liquids, *Aliivibrio fischeri*, isobole, mixture toxicity, concentration addition, independent action, co-toxicity coefficient, antagonism

## Abstract

Chirality is an important property of molecules. The study of biological activity and toxicity of chiral molecules has important theoretical and practical significance for toxicology, pharmacology, and environmental science. The toxicological significance of chiral ionic liquids (ILs) has not been well revealed. In the present study, the enantiomeric joint toxicities of four pairs of chiral ILs 1-alkyl-3-methylimidazolium lactate to *Allivibrio fischeri* were systematically investigated by using a comprehensive approach including the co-toxicity coefficient (CTC) integrated with confidence interval (CI) method (CTCICI), concentration-response curve (CRC), and isobole analysis. The direct equipartition ray (EquRay) design was used to design five binary mixtures of enantiomers according to molar ratios of 1:5, 2:4, 3:3, 4:2, and 5:1. The toxicities of chiral ILs and their mixtures were determined using the microplate toxicity analysis (MTA) method. Concentration addition (CA) and independent action (IA) were used as the additive reference models to construct the predicted CRC and isobole of mixtures. On the whole, there was an enantioselective toxicity difference between [BMIM]D-Lac and [BMIM]L-Lac, and [HMIM]D-Lac and [HMIM]L-Lac, while no enantioselective toxicity difference was observed for [EMIM]D-Lac and [EMIM]L-Lac, and [OMIM]D-Lac and [OMIM]L-Lac. Thereinto, the enantiomer mixtures of [BMIM]D-Lac and [BMIM]L-Lac, and [HMIM]D-Lac and [HMIM]L-Lac presented antagonistic action, and the enantiomer mixtures of [EMIM]D-Lac and [EMIM]L-Lac, and [OMIM]D-Lac and [OMIM]L-Lac overall presented additive action. Moreover, the greatest antagonistic toxicity interaction occurred at the equimolar ratio of enantiomers. Based on these results, we proposed two hypotheses, (1) chiral molecules with enantioselective toxicity difference tended to produce toxicity interactions, (2) the highest or lowest toxicity was usually at the equimolar ratio and its adjacent ratio for the enantiomer mixture. These hypotheses will need to be further validated by other enantiomer mixtures.

## 1. Introduction

Chirality is the geometric characteristic of a molecule being not superimposable on its mirror image formed by inversion through a point by pure rotation and translation. Chiral molecules with a single chiral center have two stereochemical arrangements, which have identical physicochemical properties, but can differ in biological activity and toxicity [1]. Biological systems are also chiral environments composed of biological macromolecules (such as enzymes), which interact differentially with chiral small molecules. Chiral enantiomers enter the organism and are identified and matched by the chiral environment as different molecules. Enantiomers usually have stereoselectivity in pharmacokinetics, pharmacodynamics, and drug metabolism (PK/PD/DM), and toxicology [2]. 

The pharmacology and toxicology significances of chiral drugs and chiral pesticides have been widely recognized. For example, the stereoselective potencies and relative toxicities of coniine enantiomers and racemate were evaluated to human rhabdomyoma cells and mice [3]. The efficacy, toxicity, pharmacokinetics, and in vitro metabolism of the enantiomers and racemate of ifosfamide were studied in mice [4]. The stereoselective metabolism, pharmacokinetics, pharmacodynamics, and toxicity of vasicine enantiomers were studied in vitro and in vivo [5]. Asymmetric dimethylarginine and its enantiomer symmetric dimethylarginine were associated with chronic kidney disease and other cardiovascular risks [6]. The enantiomers of naproxen, ibuprofen, ketoprofen, and flurbiprofen were evaluated in bioassays with bacteria, algae, and fish cells [7]. The enantioselective toxicity difference was observed for dinotefuran, *S*-dinotefuran was 41.1 to 128.4 fold more toxic than *R*-dinotefuran to honeybee, whereas *R*-dinotefuran exhibited comparative insecticidal activities (1.7–2.4 times) compared to the racemic mixtures [8]. Systematic assessments for four stereoisomers of propiconazole with two chiral centers were performed, including absolute configuration, stereoselective bioactivity, and toxicity [9]. The enantioselectivity of isocarbophos were studied in rice cultivation, including bioactivity, toxicity, and environmental fate [10]. The activity, toxicity, molecular docking, and environmental effects of three pairs of imidazolinone herbicides enantiomers were studied [11]. Chiral herbicide dichlorprop can induce the chiral macroaggregates structural change of light-harvesting chlorophyll a/b pigment-protein complexes, which was associated with the enantioselective toxicity to *Scnedesmus obliquus* [12]. 

Although enantioselective toxicity difference of chiral pesticides has been received fundamental understanding during the past few decades, how coexisting enantiomers interacting with each other during their toxicity action remained largely unknown. Various methods have tried to understand the toxic interaction between enantiomers. Metabolomics were used to study the metabolic perturbations and toxic effects of *rac*-metalaxyl and metalaxyl-M in mice using NMR and UPLC-MS/MS [13]. Molecular docking methods based on computational chemistry were used to compare the binding affinities of enantiomer pairs to the protein target [14]. The toxic unit (TU) method was employed to evaluate the joint additive toxicity of isocarbophos enantiomer to *Daphnia magna* [15]. Isobole analysis was used to evaluate the synergistic action between the enantiomers of tramadol [16]. Although both TU and isobole models can be attributed to the CA model, CA and IA as the mainstream additive reference models for the prediction and assessment of mixture toxicity at present [17], have not been widely and deeply applied in enantiomer combined toxicity studies. In the present study, the methods of CA, IA, isobole, and CTCICI [18] were used to comprehensively analyze the toxic interaction of enantiomer mixtures.

ILs are a group of organic salts that are liquid at room temperature [19]. The application areas of ILs included catalysis, extraction, synthesis, dissolution, food science, and so on [20]. With the development of industry and the needs of the society, chiral ILs have also achieved considerable development [21]. Chiral ILs have been used in optical resolution, asymmetric synthesis, chiral stationary phase in chromatography, and chiral selectors [22]. To date, most of the reported chiral ILs were those based on chiral cations, only a very limited number of chiral ILs contained chiral anions [23]. The toxicological significance of chiral ILs has not been well revealed. Although, many studies have reported the biological toxicities of ILs to enzymes, bacteria, algae, mammalian cells, plants, invertebrates, and vertebrates [24,25,26,27,28,29,30], However, most of these ILs studied were non-chiral. Chiral ILs may also exhibit enantioselective toxicity difference. For example, for 1-alkyl-3-methylimidazolium lactate, there was a distinct difference between the toxicities of [EMIM]L-(+)-Lac and [EMIM]D-(−)-Lac toward green algae *Scenedesmus obliquus* and *Euglena gracilis* [31]. Chiral ILs 1-alkyl-3-methyl imidazolium tartrate can cause enantioselective oxidative stress to *Scenedesmus obliquus*, and the toxicity of [RMIM]L-(+)-tartrate treatment was greater than [RMIM]D-(-)-tartrate with enantioselectivity [32]. However, the toxicity interaction of chiral ILs enantiomers was still not entirely clear and has not been systematically studied up to now.

In the present study, we selected four pairs of chiral ILs composed of imidazolium cation and lactate anion as the enantiomer components. They were [EMIM]D-Lac and [EMIM]L-Lac, [BMIM]D-Lac and [BMIM]L-Lac, [HMIM]D-Lac and [HMIM]L-Lac, and [OMIM]D-Lac and [OMIM]L-Lac. Their specific information is shown in the section of Materials and Methods. Among them, the imidazole cations were not chiral. While lactate anion has two enantiomers of L-lactate and D-lactate, and the human body can only metabolize the L-lactic acid [33]. We want to know how coexisting chiral ILs enantiomers interact with each other in the toxicity. Such considerations prompt us to initiate this study, which aims (1) to investigate the enantioselective toxicity difference for chiral ILs, (2) to investigate the enantiomeric toxicity interaction of chiral ILs, and (3) to determine whether there was some relationship between enantioselective toxicity difference and enantiomeric toxicity interaction.

## 2. Results and Discussion

### 2.1. Single Enantiomer Toxicity

**Figure 1:**
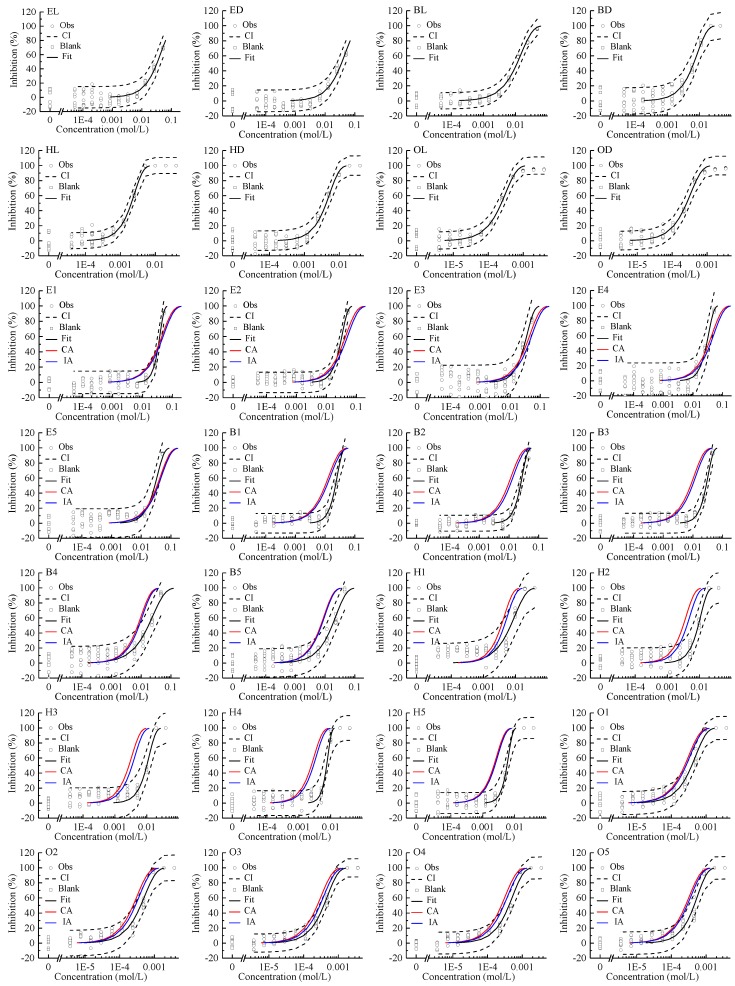
Concentration–response curves of single enantiomer and their mixtures of four pairs of chiral ionic liquids inhibiting *Allivibrio fischeri*. Note: Square: blank control; Circle: observed data; Black dashed line: confidence interval; Black solid line: Weibull model fit; Red line: Concentration addition (CA) prediction; Blue line: independent action (IA) prediction.

All of the studied enantiomers inhibited *Allivibrio fischeri* (AVF) in a concentration-dependent manner, with log-sigmoidal CRC for the four pairs of chiral ILs shown in Figure 1. The concentration of the stock solutions (C_0_) and following diluted solutions (C_1_–C_11_) of chiral ILs and their mixtures were shown in Table S1. The regression models and the estimated parameters of the toxicity of single enantiomer to AVF are summarized in Table 1. The CRCs can be fitted by the two-parameter Weibull function with RMSE < 0.09 and *R*^2^ > 0.91. The variability of the blank control in the test was controlled within ±20%. The indicators of effect concentration EC_80_, EC_50_, and EC_30_ are shown in Table 1. According to these indicators, the toxicity order of single enantiomers was OL ≥ OD > HL > HD > BD > BL > EL ≥ ED. With the increase of the number of carbon atoms in the alkyl chains of the imidazolium cations, the toxicity of chiral ILs increased gradually. The EC_50_ of EL was 143 times that of OL, and the EC_50_ of ED was 137 times that of OD. Stock et al. also reported that ILs with long alkyl chains showed higher AVF inhibitive toxicity [34].

**Figure 2:**
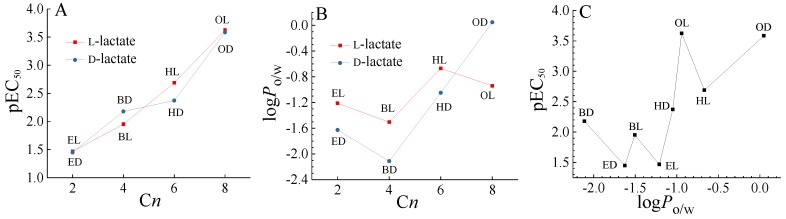
(**A**) Relationship between the toxicity (pEC_50_) and the number of carbon atoms (C*n*) in the alkyl chains of the imidazolium cations of single ionic liquids (ILs), (**B**) relationship between the n-octanol/water partition coefficient (log*P*_o/w_) and the C*n* of single ILs, and (**C**) relationship between the pEC_50_ and the log*P*_o/w_ of single ILs.

Previous studies have demonstrated that the enantiomers of chiral pesticides have different biological toxicities [9]. In the present study, the EC_50_ of BL was 1.7 times that of BD, and the EC_50_ of HD was 2.1 times that of HL. BL and BD, and HD HL showed enantioselective toxicity to AVF. While enantioselective toxicity difference was not observed for ED and EL, and OD and OL to AVF as shown in Figure 2A. Chen et al. reported the enantioselective toxicity of these four pairs of chiral lactate ILs to *Scenedesmus obliquus*, the EC_50_ value of EL was twice that of ED to algae, while no enantioselective toxicity difference was found for L-lactic acid and D-lactic acid [31]. Therefore, the enantioselective toxicity difference of the chiral ILs enantiomers with lactate anion should be attributed to the interaction between anions and cations. 

The n-octanol/water partition coefficient (log*P*_o/w_) was an important parameter that can be used to simulate ILs diffusion from the aqueous phase to the bacterial cell membrane, and was also associated with the chemical toxicity [35]. We determined the log*P*_o/w_ of these chiral ILs as shown in Figure 2B and Table S2. It can be seen that basically the log*P*_o/w_ of L-lactate was greater than the log*P*_o/w_ of D-lactate, except for the octyl IL in the opposite order. Meanwhile, compared with ethyl, hexyl, and octyl ILs, the log*P*_o/w_ of butyl IL was the smallest, indicating that the trend of butyl IL participating in water was the largest relative to n-octanol. Normally, with the increase of the length of alkyl chain, the toxicity and lipophilicity of ILs were increased as shown in Figure 2A,B. However, the relationship between lipophilicity (log*P*_o/w_) and toxicity (e.g., pEC_50_) was generally an inverted U-shaped, as shown in Figure 2C and the reference [36]. Therefore, there may be some balance, resulting in enantioselective toxicity difference in the imidazole lactate ILs with an intermediate number of carbon atoms. Chen et al. also observed that chiral ILs with greater carbon chain lengths no longer exhibited enantioselectivity, due to changes in the toxicity weightings of the cations [31]. Furthermore, the mechanisms of enantioselective toxicity difference of chiral ILs at the molecular and cellular levels will need further study to be elucidated [32].

ILs were different from ordinary neutral molecules. ILs can be regarded as a mixture of anions and cations. Stolte et al. proposed that the toxicity of single ILs can be calculated based on their anion and cation toxicity using the CA model [37]. Previous studies showed that the strong electrostatic interactions between an achiral cation and a chiral anion can result in the transfer, induction, and amplification of chiral information [38,39]. Such ion-pairing effects between achiral imidazolium cation and chiral lactate anion may induce the enantioselective toxicity difference.

### 2.2. Enantiomer Mixture CRC and CTC

The mixture CRCs predicted by CA and IA together with the experimental data and the fitted curves were integrated and displayed in Figure 1. These observed CRCs can also be depicted by the Weibull function. In all cases, the *R*^2^s were greater than 0.85 and the RMSEs less than 0.13.

The EC_80_, EC_50_, and EC_30_ values for enantiomer mixtures are listed in Table 1. Using the EC*_x_*_,*i*_, EC*_x_*_,mix_, and *P_i_*, the CTC of IL enantiomer mixtures can be obtained as shown in Table 2. According to the CTCICI method [18], at 30%, 50%, and 80% effect levels, the mixtures of BD and BL, HL, and HD, and OD and OL overall presented antagonistic action, except for the additive action of H2, H3 at 80% effect, H1 at 50% effect, B4, B5, H1, O1, O2, and O4 at 30% effect. While ED and EL mixtures presented additive action at 30% and 50% effect levels, and presented synergistic action at 80% effect level except for the additive action of the E3 mixture. Theoretically, CTC [40] was the deformation expression of CA, and the reciprocal form of the combination index [41]. Therefore, CTC only reflected the judgment result of CA on toxic interaction [42]. The advantages of CTC were simple, intuitive, quantitative, and widely used. Although in most cases, the difference between the CA and IA predictions were small for an assessment of mixture toxicity [43]. However, sometimes this difference can be too big to ignore [44,45]. The next step was to further study the toxic interaction of these four pairs of enantiomer mixtures from the perspective of CRC based on CA and IA in combination with CI.

Comprehensively speaking, the predicted curves of CA and IA were above the observed CRC CI for BD and BL, and HL and HD mixtures, these two pairs of enantiomers were antagonistic action. While the predicted curves of CA and IA were within the observed CRC CI for ED and EL, and OD and OL mixtures, so these two pairs of enantiomers were additive action, except that the mixtures of ED and EL were synergistic action at the 80% effect level. Single enantiomer toxicity analysis indicated that enantioselective toxicity differences were observed for BD and BL, and HL and HD mixtures, and that were not observed for ED and EL, and OD and OL mixtures. This generated a hypothesis that chiral molecules with enantioselective toxicity difference tended to produce toxicity interaction. 

### 2.3. Enantiomer Mixture Toxicity Assessment Based on Isobole

**Figure 3:**
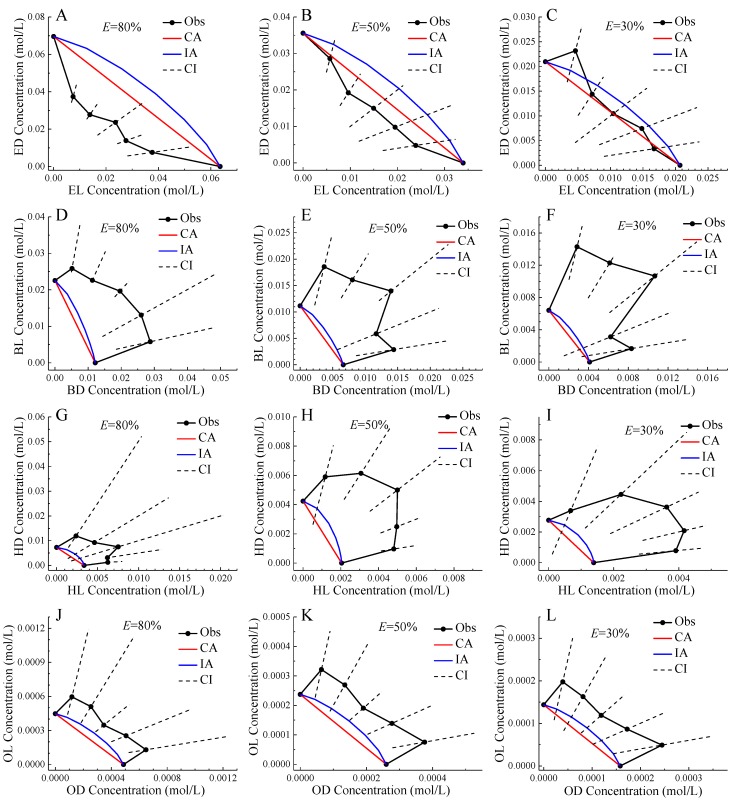
Isoboles of binary mixtures of enantiomers of four pairs of chiral ionic liquids to *Allivibrio fischeri* at 80%, 50%, and 30% effect levels. Note: Black point: observed data; Black solid line: observed isobole; Black dashed line: confidence interval; Red line: CA isobole; Blue line: IA isobole; except for the two boundary points of the black solid line, the remaining five points in line order from left to right correspond to the enantiomer molar ratios of 1:5, 2:4 , 3:3, 4:2, and 5:1.

Figure 3 showed the isoboles of four pairs of chiral ILs mixtures at 30%, 50%, and 80% effect levels. In all situations, the CA isoboles were below the IA isoboles, which was the reflection of CA CRC above IA CRC. In general, the observed isoboles of the mixtures of ED and EL, and OD and OL were relatively close to the predicted isoboles of CA and IA, and the CI of the observed isoboles can basically contain the CA or IA isobole, so the two pairs of enantiomers were additive action. The only exception was ED and EL mixture presenting synergistic action at 80% effect level. In the Section 2.2, mixtures of OD and OL were judged to show antagonistic action. However, based on IA, these mixtures were determined to be additive. Therefore, CA and IA should be used in combination to comprehensively judge the toxicity interaction of mixtures to avoid qualitative error [18]. On the other hand, the observed isoboles of the mixtures of BD and BL, and HD and HL deviated from the predicted isoboles of CA and IA far upward, and the CI of the observed isoboles basically cannot contain the CA or IA isobole, so these two pairs of enantiomers showed antagonistic action, and the mixture with molar ratio 1:1 generally had the largest deviation trend indicating the greatest toxic interaction.

It was generally accepted that CA was applicable to similar acting chemicals and IA was applicable to dissimilar acting chemicals [46]. It should be more likely that enantiomers were applicable to CA, but our results indicated that IA was closer to mixture observed isobole than CA. Therefore, the question was aroused as to whether CA and IA were related to the mechanism of action (MoA). Our results supported that CA and IA were both only the additive reference models, which were not applicable to associate with the MoA. The additivity assumption was merely a working concept and did not necessarily reflect the reality [47].

In the present study, we used three methods of CTCICI, CRC, and isobole to evaluate the toxic interaction of enantiomers of chiral ILs. To date, there were few examples of evaluating enantiomeric mixture effects based on isobole [16]. Most of studies regarding enantiomer mixture toxicity evaluation were based on statistical judgments by comparing with the toxicity of single enantiomers [48]. The advantage of this approach was that it was simple to operate and did not require the introduction of an additive reference model. In particular, when the effect concentration of enantiomer mixture was greater than or less than the corresponding effect concentration of enantiomer components, the mixture would show antagonistic or synergistic action [49]. In theory, this was still based on the CA principle. As shown in Table 1, all the mixtures of BD and BL, HD and HL, and OD and OL at 30%, 50%, and 80% effect for all molar ratios, the enantiomer mixture effect concentrations (EC*_x_*_,mix_) were all greater than single enantiomer effect concentrations (EC*_x_*_,*i*_), which indicated that these mixtures were theoretically antagonistic based on CA. Only the mixture of ED and EL at all molar ratios, the mixture 80%-effect concentrations (EC_80__,mix_) were all smaller than the enantiomer 80%-effect concentrations (EC_80__,*i*_), which indicated that these mixtures were theoretically synergistic based on CA. In these cases, toxic interactions can be determined even without the use of statistical judgments, additive models, or isobole. However, when the mixture effect concentration was within or not far from the enantiomer effect concentration, it was necessary to use the additive model or isobole in combination with the CI. Comparatively speaking, isobole had the natural applicability, comprehensiveness, and accuracy in judging enantiomeric toxic interactions.

### 2.4. Relationship between Mixture Toxicity and Enantiomer Concentration Proportions

**Figure 4:**
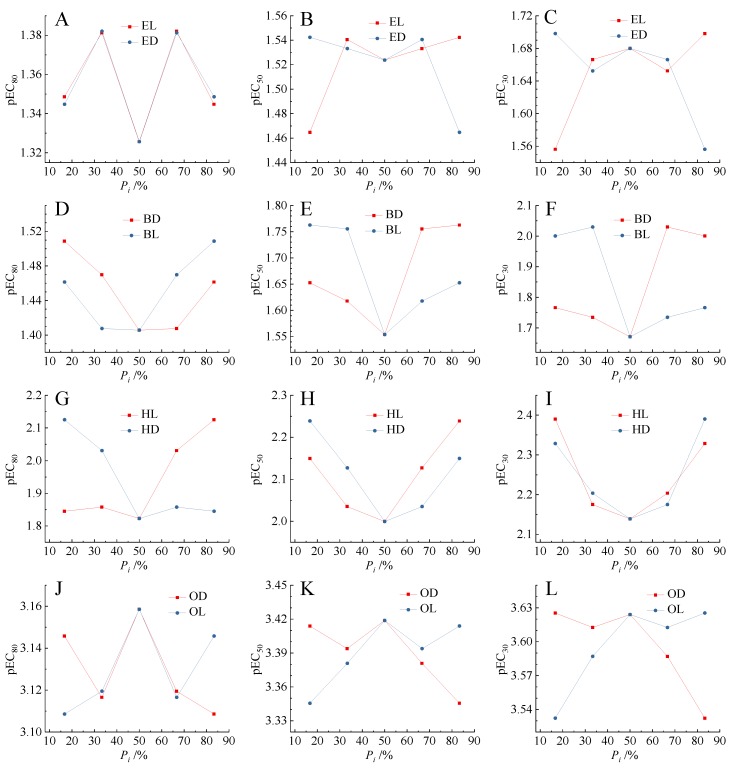
Relationship between the toxicity (pEC_x_) of enantiomeric binary mixtures and the concentration proportion (*P_i_*) of enantiomer components. (**A**–**C**) Mixtures of [EMIM]L-Lac and [EMIM]D-Lac; (**D**–**F**) mixtures of [BMIM]D-Lac and [BMIM]L-Lac; (**G**–**I**) mixtures of [HMIM]L-Lac and [HMIM]D-Lac; (**J**–**L**) mixtures of [OMIM]D-Lac and [OMIM]L-Lac.

Previous studies indicated that there was biphasic relationship between the binary mixture toxicity and the concentration proportion (*P_i_*) of components [18]. A pair of enantiomers of chiral molecules can form the natural binary mixtures. It can be seen that in Figure 4, there were two pairs of relatively obvious U-shaped relationship between the *P_i_* of components and the toxicities (pEC_30_, pEC_50_, pEC_80_) of mixtures of BD and BL, and HL and HD. There were two pairs of inverted U-shaped relationships between the *P_i_* of components and the toxicities (pEC_30_, pEC_50_, pEC_80_) of mixtures of EL and ED, and OD and OL. This phenomenon basically conformed to the climax hypothesis proposed by Lin et al. [50]. The climax hypothesis concluded that there was a climax at the equitoxic ratio when plotting the toxic ratios of individual chemicals in mixtures versus their joint effects [51]. 

In the present study, we deliberately designed the enantiomer mixtures according to their molar ratio, and found that the highest or lowest toxicity point was usually at the equimolar ratio, especially for the enantiomers with differential toxicity. Therefore, for the enantiomer mixture, the climax hypothesis was reduced to a new form that the highest or lowest toxicity was usually at the equimolar ratio. To show the difference, we called this type of Climax hypothesis the Crown hypothesis according to the shape of the isobole. This new improved hypothesis needed to be tested by other enantiomeric binary mixtures.

### 2.5. Implications

The equimolar ratio corresponding to the greatest antagonism may reflect a certain mechanism in itself. When the ratio of enantiomers was greatly different, the one with a large proportion played a dominant role, the antagonism was also small accordingly. However, when the proportion of the two enantiomers was the same, the two had even influence and produced the greatest antagonism. More quantitative and accurate interpretation may need to apply the molecular simulation to a specific protein molecular target, such as the photobacterium luciferase.

Our results showed that the antagonistic toxicity interaction was strongest when the enantiomeric mixture was at the equimolar ratio. Raffa et al. also observed that the mixture of the tramadol enantiomers with the ratio (−)/(+) = 1/1 produced the strongest synergistic action to mice for the antinociception effect expressed as inhibition of acetylcholine-induced abdominal constriction [16]. When mixtures were presented in equimolar ratio and its adjacent ratio, the maximum medicinal interaction can also be observed in drug synergies. In fact, the ratio-dependent synergy had also been used in pharmacology. Ribavirin and disulfiram in molar ratio 2:1 presented the maximal antibacterial synergy against methicillin-resistant *Staphylococcus aureus* proliferation [52]. Indacrinone was a diuretic, the (−) isomer had diuretic effect with increasing the uric acid levels in blood, while the (+) isomer can promote the excretion of uric acid. It was possible to improve the therapeutic effects of indacrinone by manipulation of the enantiomer ratio, such as (−)/(+) = 1/4 [53]. However, whether the equimolar ratio and its adjacent ratio presenting the maximum toxic interaction was universal required further verification.

For the ILs toxicity, it was generally accepted that the cations played a major role and were more important than anions. Our results suggested that the interaction between cations and anions may actually be more important than single ions. Our results also showed that the toxicity of octyl ILs was two orders of magnitude greater than that of ethyl ILs, while different configurations of lactate ILs had no fixed order of toxicity. To minimize the environmental risk, the chiral ILs with short alkyl chains and enantioselective toxicities should be taken into consideration [31].

## 3. Materials and Methods

### 3.1. Chemicals

The IL components included 1-ethyl-3-methylimidazolium D-lactate ([EMIM]D-Lac), 1-ethyl-3-methylimidazolium L-lactate ([EMIM]L-Lac), 1-butyl-3-methylimidazolium D-lactate ([BMIM]D-Lac), 1-butyl-3-methylimidazolium L-lactate ([BMIM]L-Lac), 1-hexyl-3-methylimidazolium D-lactate ([HMIM]D-Lac), 1-hexyl-3-methylimidazolium L-lactate ([HMIM]L-Lac), 1-octyl-3-methylimidazolium D-lactate ([OMIM]D-Lac), and 1-octyl-3-methylimidazolium L-lactate ([OMIM]L-Lac). These ILs were purchased from Shanghai Chengjie Chemical Co. LTD. (Shanghai, China). The chemical structures and related information of these ILs are shown in Table 3. 

The stock solutions of ILs were separately prepared through dissolving them in the deionized water and stored in a 4 °C refrigerator. The stock solutions of IL mixtures were prepared through mixing the stock solutions of individual ILs according to their concentration ratios assigned. The concentration of the stock solutions (C_0_) and following diluted solutions (C_1_–C_11_) of chiral ILs and their mixtures were shown in the Supplementary Materials.

### 3.2. Photobacterium Toxicity Test

The photobacterium *Aliivibrio fischeri* (Strain number 1H00019) was purchased from Marine Culture Collection of China (MCCC). The culture medium consisted of 1 g KH_2_PO_4_, 4.7 g Na_2_HPO_4_·12H_2_O, 0.3 g MgSO_4_·7H_2_O, 0.5 g (NH_4_)_2_HPO_4_, 30 g NaCl, 5.0 g yeast extract powder, 5.0 g tryptone, 3.0 g glycerin, and 1000 mL water, and was adjusted to pH 6.7 ± 0.3. The AVF was grown in the culture medium at 22 ± 1 °C by shaking (120 r/min) for 8–12 h during the logarithmic growth phase until the relative light unit reached 1 × 10^6^ for the toxicity test.

The toxicities of single ILs and their mixtures were expressed as an inhibition of the AVF luminescence. According to the methods of MTA [54], IL chemicals and their mixtures with 11 concentration series in eight repeats and eight controls were arranged in a microplate. First, 100 µL water was added to eight wells in the twelfth column as blank controls, 100 µL of the solutions of IL chemicals, and their mixtures with 11 gradient concentrations according to a geometric dilution factors of 0.5 were added to the wells from the first to the eleventh column. Then, 100 μL AVF suspension was added into each well to reach the final volume of 200 μL. The relative light units (RLUs) of the AVF system exposed to single ILs and their mixtures were determined on Synergy 2 Multi-Mode Microplate Readers (BioTek Instruments, Winooski, VT, USA) with a 96-well white flat bottom microplate (Corning 3917) after 30 min of exposure at 26 ± 1 °C.

The inhibitive effect (*E* of *x*%) of individual ILs and their mixtures was calculated using Equation (1). The CRCs were fitted by Weibull function shown in Equation (2) using least squares method [55]. The goodness of fit of statistical models was evaluated by *R*^2^ and RMSE. As a quantitative measure of the uncertainty, the observation-based 95% CI was determined [56].

(1) $$E=1-\frac{L}{L_{0}}$$

(2) $$\;E=1-\exp\left(-\exp\left(a+b\;\times {\;\log}_{10}\left(C\right)\right)\right)\;$$

where *L*_0_ is the average of RLUs of controls, *L* is the average of RLUs of treatments, *E* is inhibitive effect of AVF luminescence, *C* is chemical concentration, *a* is location parameter, and *b* is slope parameter.

### 3.3. Experimental Design and Toxicity Evaluation of Mixtures

The five binary mixtures of each pair of enantiomers were designed using the EquRay design [57], every two enantiomers were mixed according to molar ratios of 1:5, 2:4, 3:3, 4:2, and 5:1. The molar ratio was chosen instead of the toxic unit ratio to construct the racemic mixture and to increase the generality of the experimental design. 

The models of CA shown in Equation (3) and IA shown in Equation (4) were used to predict the mixture effect concentration (EC*_x_*_,mix_) corresponding to the mixture x% effect, and the predicted CRC of the mixture was also presented [58]. The predicted EC*_x_*_,mix_ was multiplied by the enantiomer concentration fraction (*P_i_*) to obtain the two partial concentrations which formed a point in the two-dimensional Cartesian coordinates. These points were connected to form the mixture predicted isobole. When the CIs of mixture observed isobole were containing, above, or below the mixture predicted isobole, the mixture was judged to present additive, antagonistic, or synergistic action, respectively.

For CRC, when the mixture predicted CRC was located within the CIs of mixture observed CRC, the mixture presented additive action. When the mixture predicted CRC was located above or below the mixture observed CRC CI, the mixture presented antagonistic or synergistic action, respectively.

At the single effect point level, the toxic interactions of mixtures were evaluated using the components EC*_x_*_,*i*_, and mixture observed EC*_x_*_,mix_ and its 95% CI according to the CTCICI method developed recently [18]. The CTC were computed using Equation (5). When 100 was included in the CI of mixture CTC, the mixture presented additive action. When the CI of mixture CTC was greater or smaller than 100, the mixture presented synergistic or antagonistic action, respectively.

(3) $${\mathrm{EC}}_{x,\mathrm{mix}}=1/\overset{n}{\underset{i=1}{\sum }}\left(P_{i}/{\mathrm{EC}}_{x,i}\right)$$

(4) $$\;x\%=1-\overset{n}{\underset{i=1}{\prod }}\left(1-F_{i}\left(P_{i}\times {\mathrm{EC}}_{x,\mathrm{mix}}\right)\right)\;$$

(5) $$\;\mathrm{CTC}=100/\left({\mathrm{EC}}_{x,\mathrm{mix}}\;\times \;\overset{n}{\underset{i=1}{\sum }}\left(P_{i}/{\mathrm{EC}}_{x,i}\right)\right)\;$$

where *n* is the number of mixture components, *EC_x_*_,*i*_ is the concentration of *i*th component eliciting the *x*% effect, *EC_x_*_,mix_ is the concentration of a mixture eliciting the *x*% effect, *P_i_* is the concentration proportion of *i*th component in a mixture, *F_i_* is individual concentration-response functions, CTC is co-toxicity coefficient.

## 4. Conclusions

The toxicities of the four pairs of chiral ionic liquids (ILs) to *Allivibrio fischeri* were explored. Interestingly, two pairs of chiral ILs showed enantioselective toxicity difference, while the other two pairs of chiral ILs showed no enantioselective toxicity difference. Thereinto, the enantiomer mixtures of two pairs of chiral ILs with enantioselective toxicity difference presented antagonistic action, that without enantioselective toxicity difference overall presented additive action. Moreover, the greatest toxic interaction occurred at the enantiomer molar ratio 1:1. These results may have important implications and practical applications for chiral molecules in toxicology, pharmacology, environmental science, pesticide science, and other research fields.

## Figures and Tables

**Table 1:** Concentration–response model of single enantiomer and their binary mixtures of four pairs of chiral ionic liquids inhibiting *Allivibrio fischeri* and related parameters.

Toxicants	Molar Ratio	C_0_	*a*	*b*	*R* ^2^	RMSE	EC_30_	EC_50_	EC_80_
EL		1.05 × 10^–1^	4.178	3.093	0.913	0.075	2.07 × 10^−2^	3.39 × 10^−2^	6.35 × 10^−2^
ED		1.01 × 10^−1^	3.821	2.890	0.910	0.072	2.09 × 10^−2^	3.56 × 10^−2^	6.96 × 10^−2^
BL		9.51 × 10^−2^	5.020	2.759	0.978	0.054	6.41 × 10^−3^	1.12 × 10^−2^	2.25 × 10^−2^
BD		9.00 × 10^−2^	6.596	3.196	0.958	0.088	4.11 × 10^−3^	6.63 × 10^−3^	1.22 × 10^−2^
HL		7.92 × 10^−2^	10.19	3.925	0.988	0.053	1.38 × 10^−3^	2.04 × 10^−3^	3.35 × 10^−3^
HD		7.83 × 10^−2^	8.155	3.592	0.980	0.065	2.77 × 10^−3^	4.24 × 10^−3^	7.28 × 10^−3^
OL		7.91 × 10^−3^	10.70	3.053	0.983	0.057	1.44 × 10^−4^	2.37 × 10^−4^	4.48 × 10^−4^
OD		6.79 × 10^−3^	10.68	3.081	0.979	0.063	1.58 × 10^−4^	2.60 × 10^−4^	4.88 × 10^−4^
E1	1:5 (EL:ED)	1.02 × 10^−1^	10.26	7.255	0.938	0.074	2.78 × 10^−2^	3.43 × 10^−2^	4.48 × 10^−2^
E2	2:4 (EL:ED)	1.02 × 10^−1^	7.783	5.290	0.946	0.067	2.16 × 10^−2^	2.88 × 10^−2^	4.16 × 10^−2^
E3	3:3 (EL:ED)	1.03 × 10^−1^	6.114	4.253	0.850	0.113	2.09 × 10^−2^	2.99 × 10^−2^	4.72 × 10^−2^
E4	4:2 (EL:ED)	1.04 × 10^−1^	8.181	5.575	0.876	0.119	2.23 × 10^−2^	2.93 × 10^−2^	4.15 × 10^−2^
E5	5:1 (EL:ED)	1.04 × 10^−1^	6.210	4.264	0.882	0.096	2.00 × 10^−2^	2.87 × 10^−2^	4.52 × 10^−2^
B1	1:5 (BD:BL)	9.42 × 10^−2^	9.308	5.854	0.960	0.065	1.71 × 10^−2^	2.23 × 10^−2^	3.10 × 10^−2^
B2	2:4 (BD:BL)	9.33 × 10^−2^	8.839	5.690	0.972	0.053	1.84 × 10^−2^	2.41 × 10^−2^	3.39 × 10^−2^
B3	3:3 (BD:BL)	9.25 × 10^−2^	8.463	5.682	0.945	0.066	2.13 × 10^−2^	2.79 × 10^−2^	3.93 × 10^−2^
B4	4:2 (BD:BL)	9.16 × 10^−2^	3.885	2.422	0.855	0.111	9.34 × 10^−3^	1.76 × 10^−2^	3.91 × 10^−2^
B5	5:1 (BD:BL)	9.08 × 10^−2^	4.562	2.796	0.906	0.095	9.99 × 10^−3^	1.73 × 10^−2^	3.46 × 10^−2^
H1	1:5 (HL:HD)	7.84 × 10^−2^	5.580	2.766	0.869	0.130	4.07 × 10^−3^	7.08 × 10^−3^	1.43 × 10^−2^
H2	2:4 (HL:HD)	7.86 × 10^−2^	9.297	4.748	0.935	0.102	6.68 × 10^−3^	9.22 × 10^−3^	1.39 × 10^−2^
H3	3:3 (HL:HD)	7.87 × 10^−2^	9.164	4.766	0.927	0.102	7.26 × 10^−3^	1.00 × 10^−2^	1.50 × 10^−2^
H4	4:2 (HL:HD)	7.89 × 10^−2^	18.12	8.690	0.964	0.082	6.25 × 10^−3^	7.46 × 10^−3^	9.32 × 10^−3^
H5	5:1 (HL:HD)	7.90 × 10^−2^	16.22	7.409	0.977	0.070	4.69 × 10^−3^	5.77 × 10^−3^	7.50 × 10^−3^
O1	1:5 (OD:OL)	7.70 × 10^−3^	10.36	3.142	0.969	0.077	2.37 × 10^−4^	3.86 × 10^−4^	7.15 × 10^−4^
O2	2:4 (OD:OL)	7.50 × 10^−3^	9.944	3.038	0.960	0.085	2.44 × 10^−4^	4.04 × 10^−4^	7.65 × 10^−4^
O3	3:3 (OD:OL)	7.31 × 10^−3^	10.70	3.237	0.981	0.061	2.38 × 10^−4^	3.81 × 10^−4^	6.94 × 10^−4^
O4	4:2 (OD:OL)	7.13 × 10^−3^	10.53	3.223	0.971	0.073	2.59 × 10^−4^	4.16 × 10^−4^	7.59 × 10^−4^
O5	5:1 (OD:OL)	6.95 × 10^−3^	11.53	3.556	0.970	0.075	2.94 × 10^−4^	4.51 × 10^−4^	7.79 × 10^−4^

Note: C_0_ is stock concentration; *a* is location parameter; *b* is slope parameter; *R^2^* is coefficient of determination; RMSE is root-mean-square error; EC_80_, EC_50_, and EC_30_ are the 80%, 50%, 30%-effect concentration, respectively; all the units of C_0_, EC_80_, EC_50_, and EC_30_ are mol/L.

**Table 2:** Joint toxicity effect of enantiomer mixtures of four pairs of chiral ionic liquids to *Allivibrio fischeri.*

	E = 80%				E = 50%				E = 30%			
Mixtures	CTC	CTC_UL_	CTC_LL_	Interaction	CTC	CTC_UL_	CTC_LL_	Interaction	CTC	CTC_UL_	CTC_LL_	Interaction
E1	153	166	131	synergism	103	118	86	additivity	75	103	63	additivity
E2	162	177	131	synergism	122	149	96	additivity	97	138	78	additivity
E3	141	198	96	additivity	116	176	82	additivity	100	226	67	additivity
E4	158	179	131	synergism	118	193	73	additivity	93	166	60	additivity
E5	143	190	102	synergism	119	168	88	additivity	103	190	73	additivity
B1	64	66	43	antagonism	45	74	35	antagonism	34	48	29	antagonism
B2	52	52	38	antagonism	38	48	29	antagonism	29	45	28	antagonism
B3	40	44	36	antagonism	30	34	18	antagonism	23	41	17	antagonism
B4	37	66	20	antagonism	44	89	24	antagonism	50	201	25	additivity
B5	38	59	23	antagonism	41	68	26	antagonism	44	109	26	additivity
H1	43	69	10	antagonism	51	113	37	additivity	58	352	26	additivity
H2	38	124	13	additivity	34	48	23	antagonism	31	61	16	antagonism
H3	31	126	11	additivity	28	39	19	antagonism	25	49	20	antagonism
H4	44	46	21	antagonism	33	40	27	antagonism	27	38	23	antagonism
H5	49	50	39	antagonism	39	45	31	antagonism	32	45	27	antagonism
O1	64	86	32	antagonism	62	88	44	antagonism	62	112	41	additivity
O2	60	83	27	antagonism	61	90	40	antagonism	61	119	38	additivity
O3	67	81	44	antagonism	65	83	51	antagonism	63	97	45	antagonism
O4	62	78	33	antagonism	61	82	43	antagonism	59	100	40	additivity
O5	62	76	33	antagonism	57	76	41	antagonism	53	90	38	antagonism

Note: CTC: co-toxicity coefficient; CTCLL: the lower limit of mixture CTC CI; CTCUL: the upper limit of mixture CTC CI.

**Table 3:** Information about chemicals used in the experiment.

Chemicals	Abbreviation	Chemical Formula	Molecular Structure	Purity	Molecular Weight
[EMIM]D-Lac	ED	C_9_H_16_N_2_O_3_	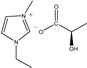	98%	200.23
[EMIM]L-Lac	EL	C_9_H_16_N_2_O_3_	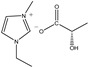	98%	200.23
[BMIM]D-Lac	BD	C_11_H_20_N_2_O_3_	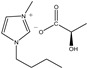	98%	228.29
[BMIM]L-Lac	BL	C_11_H_20_N_2_O_3_	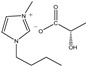	98%	228.29
[HMIM]D-Lac	HD	C_13_H_24_N_2_O_3_	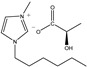	98%	256.34
[HMIM]L-Lac	HL	C_13_H_24_N_2_O_3_	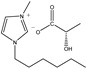	98%	256.34
[OMIM]D-Lac	OD	C_15_H_28_N_2_O_3_	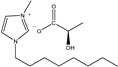	98%	284.39
[OMIM]L-Lac	OL	C_15_H_28_N_2_O_3_	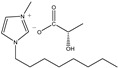	98%	284.39

## Supplementary Materials

Supplementary materials can be found at https://www.mdpi.com/1422-0067/20/24/6163/s1.
