# Supplementary material for: New Insight Regarding the Relationship Between Enantioselective Toxicity Difference and Enantiomeric Toxicity Interaction from Chiral Ionic Liquids
Source: Int J Mol Sci. 2019 Dec 6;20(24):6163. doi: 10.3390/ijms20246163 (PMC6941021; doi:10.3390/ijms20246163)
Supplement: Supplementary file 1 [file ijms-20-06163-s001.pdf]

Supporting Information for

New Insight Regarding the Relationship Between Enantioselective Toxicity Difference and Enantiomeric Toxicity Interaction from Chiral Ionic Liquids

Table S1 Molar concentrations of stock solutions (C_0) and serial diluted solutions (C_1-C_{11}) of studied chiral ionic liquids and their mixtures

	C_0	C_1	C_2	C_3	C_4	C_5	C_6	C_7	C_8	C_9	C_{10}	C_{11}
EL	1.05E-01	5.25E-02	2.63E-02	1.31E-02	6.83E-03	3.31E-03	1.63E-03	8.40E-04	4.10E-04	2.05E-04	1.05E-04	5.25E-05
ED	1.01E-01	5.05E-02	2.53E-02	1.26E-02	6.57E-03	3.18E-03	1.57E-03	8.08E-04	3.94E-04	1.97E-04	1.01E-04	5.05E-05
BL	9.51E-02	4.76E-02	2.38E-02	1.19E-02	6.18E-03	3.00E-03	1.47E-03	7.61E-04	3.71E-04	1.85E-04	9.51E-05	4.76E-05
BD	9.00E-02	4.50E-02	2.25E-02	1.13E-02	5.85E-03	2.84E-03	1.40E-03	7.20E-04	3.51E-04	1.76E-04	9.00E-05	4.50E-05
HL	7.92E-02	3.96E-02	1.98E-02	9.90E-03	5.15E-03	2.49E-03	1.23E-03	6.34E-04	3.09E-04	1.54E-04	7.92E-05	3.96E-05
HD	7.83E-02	3.92E-02	1.96E-02	9.79E-03	5.09E-03	2.47E-03	1.21E-03	6.26E-04	3.05E-04	1.53E-04	7.83E-05	3.92E-05
OL	7.91E-03	3.96E-03	1.98E-03	9.89E-04	5.14E-04	2.49E-04	1.23E-04	6.33E-05	3.08E-05	1.54E-05	7.91E-06	3.96E-06
OD	6.79E-03	3.40E-03	1.70E-03	8.49E-04	4.41E-04	2.14E-04	1.05E-04	5.43E-05	2.65E-05	1.32E-05	6.79E-06	3.40E-06
E1	1.02E-01	5.10E-02	2.55E-02	1.28E-02	6.63E-03	3.21E-03	1.58E-03	8.16E-04	3.98E-04	1.99E-04	1.02E-04	5.10E-05
E2	1.02E-01	5.10E-02	2.55E-02	1.28E-02	6.63E-03	3.21E-03	1.58E-03	8.16E-04	3.98E-04	1.99E-04	1.02E-04	5.10E-05
E3	1.03E-01	5.15E-02	2.58E-02	1.29E-02	6.70E-03	3.24E-03	1.60E-03	8.24E-04	4.02E-04	2.01E-04	1.03E-04	5.15E-05
E4	1.04E-01	5.20E-02	2.60E-02	1.30E-02	6.76E-03	3.28E-03	1.61E-03	8.32E-04	4.06E-04	2.03E-04	1.04E-04	5.20E-05
E5	1.04E-01	5.20E-02	2.60E-02	1.30E-02	6.76E-03	3.28E-03	1.61E-03	8.32E-04	4.06E-04	2.03E-04	1.04E-04	5.20E-05
B1	9.42E-02	4.71E-02	2.36E-02	1.18E-02	6.12E-03	2.97E-03	1.46E-03	7.54E-04	3.67E-04	1.84E-04	9.42E-05	4.71E-05
B2	9.33E-02	4.67E-02	2.33E-02	1.17E-02	6.06E-03	2.94E-03	1.45E-03	7.46E-04	3.64E-04	1.82E-04	9.33E-05	4.67E-05
B3	9.25E-02	4.63E-02	2.31E-02	1.16E-02	6.01E-03	2.91E-03	1.43E-03	7.40E-04	3.61E-04	1.80E-04	9.25E-05	4.63E-05
B4	9.16E-02	4.58E-02	2.29E-02	1.15E-02	5.95E-03	2.89E-03	1.42E-03	7.33E-04	3.57E-04	1.79E-04	9.16E-05	4.58E-05
B5	9.08E-02	4.54E-02	2.27E-02	1.14E-02	5.90E-03	2.86E-03	1.41E-03	7.26E-04	3.54E-04	1.77E-04	9.08E-05	4.54E-05
H1	7.84E-02	3.92E-02	1.96E-02	9.80E-03	5.10E-03	2.47E-03	1.22E-03	6.27E-04	3.06E-04	1.53E-04	7.84E-05	3.92E-05
H2	7.86E-02	3.93E-02	1.97E-02	9.83E-03	5.11E-03	2.48E-03	1.22E-03	6.29E-04	3.07E-04	1.53E-04	7.86E-05	3.93E-05
H3	7.87E-02	3.94E-02	1.97E-02	9.84E-03	5.12E-03	2.48E-03	1.22E-03	6.30E-04	3.07E-04	1.53E-04	7.87E-05	3.94E-05
H4	7.89E-02	3.95E-02	1.97E-02	9.86E-03	5.13E-03	2.49E-03	1.22E-03	6.31E-04	3.08E-04	1.54E-04	7.89E-05	3.95E-05
H5	7.90E-02	3.95E-02	1.98E-02	9.88E-03	5.14E-03	2.49E-03	1.22E-03	6.32E-04	3.08E-04	1.54E-04	7.90E-05	3.95E-05
O1	7.70E-03	3.85E-03	1.93E-03	9.63E-04	5.01E-04	2.43E-04	1.19E-04	6.16E-05	3.00E-05	1.50E-05	7.70E-06	3.85E-06
O2	7.50E-03	3.75E-03	1.88E-03	9.38E-04	4.88E-04	2.36E-04	1.16E-04	6.00E-05	2.93E-05	1.46E-05	7.50E-06	3.75E-06
O3	7.31E-03	3.66E-03	1.83E-03	9.14E-04	4.75E-04	2.30E-04	1.13E-04	5.85E-05	2.85E-05	1.43E-05	7.31E-06	3.66E-06
O4	7.13E-03	3.57E-03	1.78E-03	8.91E-04	4.63E-04	2.25E-04	1.11E-04	5.70E-05	2.78E-05	1.39E-05	7.13E-06	3.57E-06
O5	6.95E-03	3.48E-03	1.74E-03	8.69E-04	4.52E-04	2.19E-04	1.08E-04	5.56E-05	2.71E-05	1.36E-05	6.95E-06	3.48E-06

Table S2 The determined n-octanol/water partition coefficient ($\log P_{o/w}$) of single chiral ionic liquids

	EL	ED	BD	BL	HL	HD	OD	OL
$\log P_{o/w}$	-1.2	-1.6	-2.1	-1.5	-0.67	-1.0	0.048	-0.94
